# Teaching Quality Improvement: The Use of Education Theories Across the Medical Education Spectrum

**DOI:** 10.7759/cureus.26625

**Published:** 2022-07-07

**Authors:** Sugeet Jagpal, Abra Fant, Riccardo Bianchi, Andrew Kalnow

**Affiliations:** 1 Department of Medicine, Rutgers Robert Wood Johnson Medical School, New Brunswick, USA; 2 Department of Emergency Medicine, Northwestern University Feinberg School of Medicine, Chicago, USA; 3 Department of Physiology and Pharmacology, State University of New York Downstate Health Sciences University, Brooklyn, USA; 4 Emergency Medicine, OhioHealth Doctors Hospital, Columbus, USA; 5 Emergency Medicine, Ohio University Heritage College of Osteopathic Medicine, Athens, USA

**Keywords:** patient safety culture, patient safety improvement, undergraduate and graduate medical education, medical education curriculum, quality improvement projects, quality initiatives

## Abstract

It is well recognized that the principles and practices of patient safety and quality improvement (QI) need to be included in medical education. The implementation of patient safety and QI learning experiences at the undergraduate medical education (UME) and graduate medical education (GME) levels has been variable. Consistent teaching of QI across the UME-GME-continuing medical education (CME) spectrum may result in a systemic change of improved patient care and patient safety in clinical practice. We propose using education theories to frame the development of QI curricula for a longitudinal integration in medical education and clinical practice. The basic principles of four education theories, namely, reflective practice, deliberate practice, social constructivism, and organizational learning, are briefly described, and examples of their applications to QI teaching are discussed. The incorporation of education theory into the design and implementation of a longitudinal QI curriculum threaded across the UME-GME-CME spectrum may empower learners with a comprehensive and lasting understanding of QI principles and training in patient safety practice, which are essential prerequisites for the formation of a physician workforce capable of creating sustainable change in patient care.

## Introduction and background

Patient safety is an imperative focus for training quality providers. In medical education, much of the current focus on patient safety can be traced back to the case of Libby Zion and the subsequent Bell Commission, which changed duty hour rules for training in New York State in 1989, with subsequent rules being enacted by the Accreditation Council for Graduate Medical Education (ACGME) in 2003 [1]. Similar firm statements of the need to commit to patient safety have come from the 1999 Institute of Medicine publication *To Err is Human*, a scathing review of the depth of medical error in the American healthcare system estimating that up to 100,000 deaths per year may be attributed to medical error, placing iatrogenic error well within the top 10 annual causes of death [2]. Following *To Err is Human*, in 2001, the Association of American Medical Colleges recognized the need to incorporate patient safety into medical education, opening their report with the following profound statement: “Not even 50 years ago, the greatest harm when one was sick was from the disease itself. Today, sick patients are vulnerable on two fronts: one from the disease and the other from the very caregiving system in which they place their trust [3].”

However, despite the clear impetus to change the approach to patient safety within the American medical system and specifically the education and training of physicians, implementing systemic change has been fleeting. Both undergraduate medical education (UME) and graduate medical education (GME) have recognized the need to include patient safety and quality improvement (QI) initiatives into their curricula, and integration across the training spectrum is an ideal for which to strive [4,5].

Applying Kolb’s Experiential Learning Cycle is one approach to integrating QI in an immersive and longitudinal manner across the UME-GME-continuing medical education (CME) spectrum. Based on the theory that experiential learning must occur in multiple phases, including observation, conceptualization, experimentation, and experience that build upon each other, the process of QI can be refined and reinforced over time. Competency-based medical education (CBME) is a core strategy for educating physicians and focuses on outcomes and learner achievement; the need for multifaceted assessment; support of a flexible, time-independent trajectory through the curriculum; and increased accountability to stakeholders with a shared set of expectations and a common language for education, assessment, and regulation [6]. Approaching QI through an education theory and CBME lens will ideally result in improved patient safety.

To enact a system of education that is lasting and impactful, QI and patient safety should be approached in a manner similar to how the ACGME approaches its core competencies. QI is required at the undergraduate level by the Association of American Medical Colleges, at the graduate education level by the ACGME, and at the CME level by payers and health systems. There is discussion around standardizing QI education and creating competence assessments for QI, but these do not currently exist [7]. Through a holistic process of integrating this needed education, it may be possible to effect systemic change in the medical education and practice paradigm.

We propose that by integrating a longitudinal curriculum throughout the UME-GME-CME spectrum, the learner will have a more complete and lasting understanding of QI and patient safety. Incorporating education theory as described in this article may be one of the tools for embedding QI into the ongoing practice of medicine. Curricular mapping and other techniques used in CBME will be needed to make this change sustainable.

A scoping review of the available literature pertaining to medical education was performed with a discussion of the major concepts relating to QI.

## Review

Reflective practice

Reflection is a useful approach for learners to make sense of complex clinical situations [8]. It is a skill developed over time but can begin at the beginning of the trainee education, allowing learners to connect and integrate new information into existing knowledge [9,10]. Kolb describes the cycle of experiential learning to include a concrete experience, reflective observation, abstract conceptualization, and active experimentation [11]. Active experimentation involves the application of newly learned ideas to the environment, allowing for novel concrete experiences [11].

There are limited examples of incorporating reflective practice in QI education at the UME, GME, and CME levels. An example of the incorporation of a concrete experience early in medical education is a sponge retained in a dissected body in anatomy class to simulate a surgical error to be discovered by first-year medical students [12]. After this experience, students were asked to reflect on the error with an interprofessional group. This reflective observation on the importance of addressing medical errors improved students’ knowledge and attitudes on patient safety [12]. In another study, a modified structured method based on reflective practice, the Mayo Evaluation of Reflection on Improvement Tool [13], proved to be a reliable tool for assessing medical students’ reflection on safety improvement [14].

In contrast to the paucity of reflective practice in UME, there are several examples of abstract conceptualization and active experimentation in residencies, fellowships, and longitudinal programs for faculty to develop skills in QI throughout the Kolb cycle. However, there is no cohesive educational program incorporating reflective practice across the education spectrum. It seems feasible to imagine a program with graded independence in the QI process that weaves in concrete experiences with reflective observation in preclinical years with abstract conceptualization and active experimentation in the GME and CME space.

Deliberate practice

Deliberate practice is a major education theory developed in the late 20th century by K. Anders Ericsson [15] that has since been widely applied to medical education topics. In short, the theory asserts that excellence in the performance of a skill is not an innate ability but rather the result of many hours of (generally supervised) practice of a defined task associated with feedback from a coach or mentor [15]. The incorporation of feedback into the practice makes it deliberate and creates reproducible skills but is sustainable only with continued deliberate practice. To apply deliberate practice to medical fields, Ericsson additionally recommended focusing on tasks that fall into three categories along the treatment timeline: diagnosis of pathology in perceptually available stimuli, diagnosis based on the cognitive interpretation of chart-based data and information obtained in the clinical interview, and treatment of a patient through perceptual-motor performance of tasks [16].

Although many papers have looked at the acquisition of individual and team skills using deliberate practice and its corollary, mastery learning, this concept has not been extensively applied to learning or teaching QI as a whole. Organizations such as the American Heart Association have recognized that clinical outcomes are improved when training is designed with integrated, deliberate practice [17], and similar experiences are found in designing educational courses for nurse practitioners [18]. Nevertheless, most described approaches to teaching QI methodology fail to consider QI itself as a skill amenable to deliberate practice. Additionally, it is difficult to identify existing expert behavior in QI to define what mastery looks like in this domain.

It is possible to imagine that a curriculum in QI at any level of medical education would encompass deliberate practice most easily at the experiential project level. Since most QI methods are iterative, there is already a built-in process for continued practice. The necessary ingredient is simply the feedback provided by a more experienced coach or mentor figure. Although faculty time is often cited as a barrier to student or trainee engagement in QI [19,20], coaching is a fundamental component of deliberate practice, and faculty time dedicated to giving feedback should be prioritized in longitudinal QI curricula.

Social constructivism

The philosophical premise of constructivist education theories is that reality is constructed by the individual, and knowledge cannot be separated from personal experience [21]. Social constructivism builds on the foundation of both reflective and deliberate practice utilizing prior knowledge while occurring on both the individual and social levels. Social constructivism emphasizes that the learners incorporate their experiences as community members, not just individuals, to build and continually modify their world models. The learner construction of knowledge is the product of social interaction, interpretation, and understanding and critically depends on communications and interactions with others. In this educational framework, preferred approaches include contextual and experiential learning, self-directed and active learning, and collaboration and peer teaching [22].

In recent years, social constructivism paradigms have been adopted to examine simulation-based education [23,24], design competency assessments [25], evaluate interprofessional education [26,27], promote professional identity formation [28], identify the causes of migration of medical students and physicians [29], and propose transformative drivers for future medical schools [30]. We propose that a social constructivist approach can also benefit the design, adoption, and implementation of QI programs in medical education.

Reviews of best practices for high-quality curricula on QI and patient safety indicate that experiential teaching methods are included in most of these programs and that a balance between didactic and experiential learning leads to effective learning [19,31-33]. Curricula that resulted in patient care improvements and learners’ behavior all integrated an experiential component in a clinical setting. The role of informal conversations in small groups with faculty guidance during the discovery process has also been emphasized in the context of social constructivism theories [34]. In addition to curricular design, a social constructivist framework might help identify issues related to the implementation and sustainability of QI programs [35]. By applying Bourdieu’s theoretical framework to a case study, the authors identified barriers to implementation (such as finding time to schedule new QI sessions in existing curricula scheduling, recruiting faculty with topic expertise, and obtaining buy-in from stakeholders) and proposed strategies to promote these curricula in residency programs that focus on legitimizing QI and patient safety in the academic and healthcare delivery fields. We suggest that the social constructivism theory might provide a conceptual framework to develop, implement, and sustain effective QI and patient safety programs across the UME, GME, and CME spectrum.

Organizational learning

Organizational learning was initially discussed in business literature, and Gavin described a “learning organization” as one that is skilled at creating, acquiring, and transferring knowledge and modifying behaviors to reflect new knowledge and insight [36]. The elements involved in this learning organization include systematic problem-solving, experimentation with new approaches, learning from past experiences and best practices, and transferring knowledge quickly and efficiently throughout the organization. Learning organizations actively embrace the QI movement to generate ideas and collect information, and in healthcare literature, it is described in the context of QI and patient safety efforts. Learning health systems reference organizational learning principles [37].

A literature review demonstrates that organizational learning is important to medical education as graduates will not frequently work as individuals and will instead work in networks with many other professionals of varying disciplines. These organizations within which physicians work have a strong financial incentive to align practices with the latest evidence [38]. Organizational frameworks for medical centers have medical practice characteristics, including merging education and practice, making collegial, lifelong learning a part of education, and maintaining close linkages with communities through regular processes and relationships [38].

Combined approach

**Figure 1:**
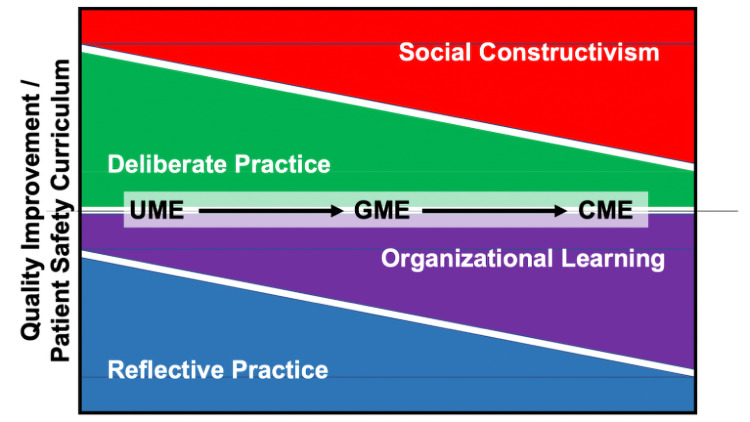
Education theories proposed for the design and integration of QI and patient safety courses into medical education programs. Different education theories can be combined in varying amounts to help frame the design, development, implementation, and assessment of QI curricula for a longitudinal integration in medical education and clinical practice. The varying bar thickness represents the extent to which each education theory could be applied at different levels of the UME-GME-CME spectrum. Various combinations of education theory can be selected to optimize the teaching of quality improvement in different programs and learning environments. Further research will need to be done to determine the optimal combination across the continuum. CME, continuing medical education; GME, graduate medical education; QI, quality improvement; UME, undergraduate medical education

Lam et al. wrote a commentary stating that residents can be trained to be “change agents” who would then have long-lasting effects on patient care throughout their careers [39]. They challenge training programs to create an environment that is open to change implementation at the institutional and health system levels. We agree that this is important, and to build a physician workforce capable of creating sustainable change, we need to integrate education theory in our QI training at all education levels and standardize the learner experience. Linking the above concepts in imagining a redesign of the education process through UME to GME to CME includes introducing the concept of working within an organization early in medical training (Figure 1). In the early phases of education, students could experience an organization that allows for lifelong learning and integration of potential practice and education.

We considered practical suggestions from the theories that emphasize reflective practice, deliberate practice, social constructivism, and organizational learning. By integrating these theories and others throughout the UME-GME-CME spectrum, the learners will more readily develop into “change agents” and be more apt to continue this work throughout their careers. Educators at these pivotal points in education should be aware of the longitudinal application of these theories, and the curriculum for QI training should be linked throughout the stages of education. The constant presence of trainees on the frontlines can be conducive to real-time assessment, reflection, and adjustment [39], but the short rotations and lack of ownership need to be addressed. By drawing attention to the CME space as a more longitudinal experience, educators can potentially guide trainees in envisioning and constructing their endeavors with a lens to the future.

When Khan et al. surveyed program directors in pulmonary and critical care regarding attitudes around quality improvement education, they found that there were substantial barriers to integrating QI at the fellowship level. Program directors did not feel that their fellows were able to conduct independent QI upon graduation [40]. The only remedy to this type of result is to build curricula that begin at the UME level and progress to the CME level. Faculty effort in mapping this curriculum for the UME level will build expertise in them as well.

We envision a standardized curriculum design for QI that focuses on empowering each student to become a change agent. This will require educators to introduce preclinical and clinical experiences that focus on problem-solving and enforce accountability for personal and team learning. The specific combination of the selected instructional and assessment methods based on different education theories may vary at different education levels and will likely have to be locally adjusted (Figure 1). As the search for the best ways to teach QI continues, additional education theories may be found to integrate or replace those discussed here. To sustainably implement this change, faculty engagement and development will be crucial. UME and GME faculty would need to communicate what each group was teaching to allow the other to reinforce, and there may need to be “just-in-time” training built-in for faculty members. Trainees from the UME and GME space could be linked in collaborative projects that would further allow for deep learning. Like any other curricular change, these processes need to be dynamic and flexible to find roots in medical education and effect change in medical practice that ensures patient safety.

## Conclusions

Competency-based medical education (CBME) is a core strategy for educating physicians, and this outcomes-oriented approach is integral to quality improvement education endeavors. We reviewed education strategies that have been used to integrate QI teaching across the medical education spectrum (UME, GME, and CME). These strategies support the development of future change agents in healthcare, which is a goal of educators in the quality improvement space. Educators must have a deep understanding of the strategies that have been successfully used across the continuum so that they can incorporate them in their future work.
